# The Families and Schools for Health Project: A Longitudinal Cluster Randomized Controlled Trial Targeting Children with Overweight and Obesity

**DOI:** 10.3390/ijerph18168744

**Published:** 2021-08-19

**Authors:** Glade L. Topham, Isaac J. Washburn, Laura Hubbs-Tait, Tay S. Kennedy, Julie M. Rutledge, Melanie C. Page, Taren Swindle, Lenka H. Shriver, Amanda W. Harrist

**Affiliations:** 1 Department of Applied Human Sciences, Kansas State University, Manhattan, KS 66506, USA; 2 Department of Human Development & Family Science, Oklahoma State University, Stillwater, OK 74078, USA; isaac.washburn@okstate.edu (I.J.W.); laura.hubbs@okstate.edu (L.H.-T.); amanda.harrist@okstate.edu (A.W.H.); 3 Department of Nutritional Sciences, Oklahoma State University, Stillwater, OK 74078, USA; tay.kennedy@okstate.edu; 4 School of Human Ecology, Louisiana Tech University, Ruston, LA 71272, USA; rutledge@latech.edu; 5 Department of Psychology, West Virginia University, Morgantown, WV 26506, USA; Melanie.Page@mail.wvu.edu; 6 Department of Family and Preventive Medicine, University of Arkansas for Medical Sciences, Little Rock, AR 72205, USA; TSwindle@uams.edu; 7 Department of Nutrition, University of North Carolina Greensboro, Greensboro, NC 27412, USA; lhshrive@uncg.edu

**Keywords:** child obesity, child overweight, randomized controlled trial, intervention, family, school

## Abstract

This cluster randomized controlled trial aimed at overweight and obese children compared three treatments. Two psychoeducation interventions for parents and children were conducted: Family Lifestyle (FL) focused on food and physical activity; Family Dynamics (FD) added parenting and healthy emotion management. A third Peer Group (PG) intervention taught social acceptance to children. Crossing interventions yielded four conditions: FL, FL + PG, FL + FD, and FL + FD + PG—compared with the control. Longitudinal BMI data were collected to determine if family- and peer-based psychosocial components enhanced the Family Lifestyle approach. Participants were 1st graders with BMI%ile >75 (*n* = 538: 278 boys, 260 girls). Schools were randomly assigned to condition after stratifying for community size and percent American Indian. Anthropometric data were collected pre- and post-intervention in 1st grade and annually through 4th grade. Using a two-level random intercept growth model, intervention status predicted differences in growth in BMI or BMI-M% over three years. Children with obesity who received the FL + FD + PG intervention had lower BMI gains compared to controls for both raw BMI (B = −0.05) and BMI-M% (B = −2.36). Interventions to simultaneously improve parent, child, and peer-group behaviors related to physical and socioemotional health offer promise for long-term positive impact on child obesity.

## 1. Introduction

Rates of obesity in children remain high [1], and the associated health consequences are a major public health concern [2]. Well-developed literatures document a variety of family and other social influences associated with children’s achieving and maintaining a healthy weight. These include influences proximal to child weight-related behaviors, such as the availability of healthy/unhealthy food options, parent strategies to encourage healthy eating, opportunities and encouragement to engage in physical activities, and parent modeling of healthy behaviors [3]. In addition, recent findings have documented the association between more distal family and peer influences and child healthy weight. Family variables related to child healthy weight include family support, general authoritative parenting style, and parental warmth and acceptance [4,5]. Children with obesity and overweight are more likely to experience difficulties in peer relationships, including heightened risk for weight-related bullying [6], peer neglect or rejection [7], and loneliness [8]. Social isolation may limit child activity levels, and loneliness and other internalizing emotions are associated with increased risk for emotional eating [8], compounding weight-related problems. To improve intervention effectiveness, it may be important for treatment efforts to include a focus on social contexts of primary importance to children, namely family and school contexts in addition to the more traditional focus on just nutrition and physical activity.

### 1.1. Conceptual Model: Families and Schools as Contexts for Intervention

The conceptual framework guiding the intervention is the Inter- and Intra-Personal Risk (IIPR) model of obesity in children [9]. The central propositions of the IIPR model are that (a) the unhealthy eating and sedentary behaviors that increase risk of obesity in children occur primarily within interpersonal contexts (e.g., the family and school peer group), and (b) child intrapersonal factors (e.g., self-regulation, negative affect) mediate the association between what is happening in the child’s interpersonal contexts and the development or maintenance of weight problems.

#### 1.1.1. Family Context

The 2017 US Preventive Services Task Force statement [10] and others [11,12] have recommended that child obesity interventions take a family-based approach, utilize behavior-change, and include multiple components targeting diet quality and physical activity. Interventions focusing on general parenting, in addition to parenting related to diet quality and exercise, appear to improve and extend treatment outcomes [13,14,15]. However, research examining long-term weight outcomes after completion of treatment is currently needed to assess long-term benefits [10].

#### 1.1.2. School Context

Schools offer another important context for child obesity interventions. The Institute of Medicine [16] describes school as a “gateway to healthy weights” (para. 2), because it is a key place where children can learn about nutrition, eat healthy foods, and exercise. In the last 20 years, the number of obesity interventions conducted in schools has multiplied. Meta-analyses of school-based RCTs and clinical controlled trials, e.g., [17], have found that these programs tend to have small, short-term positive effects on obesity, but there is a need for longer-term follow up. School-based interventions frequently target dietary behaviors, nutrition knowledge, and/or physical activity and sometimes involve parents to support lifestyle changes e.g., [18]. Arguably, the school social atmosphere has not been targeted to the same extent, even though children with obesity often experience teasing, bullying, and ostracism that could exacerbate their weight problems via social isolation and emotional eating [7].

### 1.2. Current Study

The purpose of this study was to test the effectiveness of a novel multi-arm intervention targeting proximal (food-related parenting and child healthy eating and activity) and distal (family and peer contexts) factors on child weight status over four years among first grade children in a rural mid-south area of the United States. Families were randomly assigned to the control group or one of four treatment conditions made up of different combinations of three treatment components. The three treatments were designed to target inter- and intra-personal risk factors for children already at-risk for obesity as they began their schooling. The three treatments’ components were Family Lifestyle (diet quality and exercise), Family Dynamics (authoritative parenting and child emotion regulation), and Peer Group (peer acceptance). Intervention conditions were designed to slow the rate of weight gain for children in the overweight and obese weight groups. We hypothesized that treatments including the intervention component FL by itself or in combination with the other components (FL, FL + FD, and FL + PG) would be associated with smaller gains in child BMI relative to controls. Further, we hypothesized that the intervention components would have a cumulative effect such that receiving all intervention components, (FL + FD + PG), would be associated with the smallest BMI gain. Finally, we were guided by the following research question: Will intervention components be associated with maintenance of healthy weight in children with BMI between the 75th and 85th BMI percentile (i.e., non-overweight, at-risk group)?

## 2. Materials and Methods

### 2.1. Participants

#### 2.1.1. Recruitment and Randomization

The Families and Schools for Health (FiSH) Project included a six-month intervention with three years of follow-up anthropometric assessments. Thirty-seven rural schools within a 90-mile radius of the researchers’ university were approached; 8 refused, and 29 agreed to participate. All schools where both superintendents and principals agreed to participate were included in the study. Intervention and control groups were created through a stratified random sampling procedure. Schools were stratified according to community size (above/below 10,000) and percent population of American Indian children (above/below 20%). Schools were then assigned to treatment and control conditions within the high and low groups using a random number table and electronic coin flips. The five conditions were: FL (Family Lifestyle, focusing on diet and activity), FL + FD (adding Family Dynamics focusing on psychoeducation about parenting and child socioemotional functioning), FL + PG (adding Peer Group intervention focusing on social acceptance in the classroom to FL), FL + FD + PG (adding Peer Group intervention to FL + FD), and control. After the school conditions were assigned, the researchers met with 1st grade teachers at each school to obtain consent.

All families with a 1st grade child (ages 6–7) in consented schools were invited to participate. Parents were recruited at kindergarten graduations, 1st-grade registration, and back-to-school events, as well as via letters in children’s backpacks. Families were recruited into a “healthy lifestyles” program and children were told the researchers wanted “to learn more about their eating habits.” Parents provided written informed consent, and children provided assent before participating in the study each year. Cohort 1 children who were attending intervention schools and who were assessed by the research team as being obese or at-risk for obesity (75th percentile or higher for BMI) were invited to participate in a family intervention (FL or FL + FD). To reduce stigma of participation, all Cohort 2 children not in control schools were invited to participate in a family intervention. All 1st graders at the PG schools participated in the PG intervention (because it was a classroom-level program).

Participants represented two cohorts of children, with Cohort 2 joining the study a year after Cohort 1. Wave 1 data collection began when children were mid-way through 1st grade; Wave 2 data were collected at the end of 1st grade, following intervention completion. Follow-up data (Waves 3–5) were collected annually in the springs of 2nd, 3rd, and 4th grades. The trial was registered on clincaltrials.gov (NCT02659319).

#### 2.1.2. Sample Characteristics

The sample included all participants from both cohorts who were at the 75th percentile or higher for BMI-for-age at Wave 1 (pre-intervention). This resulted in 538 children (278 boys and 260 girls) from 29 rural schools in 20 towns, with all but two towns having a population <10,000. The average proportion of children in the sample who were on free or reduced-price lunch—a proxy for poverty at the school-level—was 65%.

A mean of 71.4 children per school participated, with a minimum of 9 and a maximum of 203. On average, 3.9 assessments were conducted per child across 4 years (range = 1–5). The sample largely identified as European American (72.0%); however, a sizeable proportion identified as American Indian (18.5%). Table 1 presents demographic characteristics for the intervention and control groups; no significant differences between groups were found. In Wave 5, 51.1% (246/457) and 36.6% (29/81) of the intervention and control groups, respectively, were retained.

**Figure 1:**
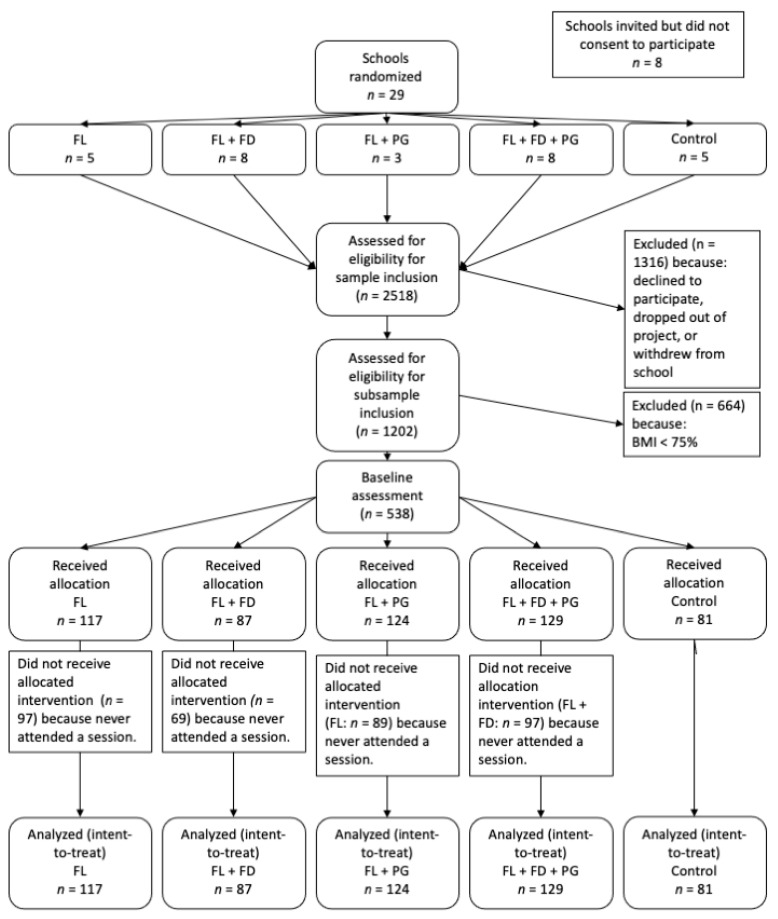
Study Recruitment Flow Diagram.

The sample was split fairly evenly among BMI-for-age percentile groups: 27.3% at-risk-but-non-overweight [19] (>75th percentile and <85th percentile), 37.7% overweight (>85th percentile and <95th percentile), and 34.9% obese (>95th percentile) at Wave 1 (See Table 2 for group by condition percentages). Regarding intervention condition assignments, 21.8% were FL; 16.2% were FL + FD; 23.1% were FL + PG; 24.0% were FL + FD + PG; and 15.1% were control. Taken together, 47.0% were allocated to receive at least PG; 84.9% were allocated to receive at least FL; and 40.2% were allocated to receive at least FD (see Figure 1 Study Recruitment Flow Diagram).

### 2.2. Procedures and Measures

Children’s height and weight were assessed at each wave in order to calculate BMI-for-age percentile using the 2000 CDC Growth charts [20]. Each child’s height was measured by the research team at school twice to the nearest 0.2 cm using a portable board (Shorr Productions, Olney, MD, USA). If the two measurements were not within +0.3 cm, then height was measured a third time. The average of the height measurements was the value used in analysis. Weight was determined to the nearest ±0.2 pounds using a portable digital scale (Tanita Electronic Scale, BWB-800). Baseline BMI-for-age percentile was used to group children into three weight status groups (hereafter referred to as BMI%ile group): obese (BMI ≥ 95%ile), overweight (85%ile ≤ BMI < 95%ile), and at-risk but not overweight (75%ile ≤ BMI < 85%ile, [21]). As noted above, children below the 75%ile were excluded from analyses. Two different BMI outcomes were operationalized in the current investigation. The first was raw BMI log transformed for skew, hereafter referred to as raw BMI. The second was log percent distance from median BMI, hereafter referred to as BMI-M% [22], calculated as 100 × ln (BMI/median BMI). BMI-M% was defined as the percent difference of raw BMI from a participant’s age and sex population median BMI. These BMI measures were chosen due to their demonstrated strengths in assessing changes in adiposity across time for children differing in levels of adiposity [23].

### 2.3. Interventions

Factors targeted by the family and school components of the intervention were based on variables identified in the investigators’ IIRP model of risk for obesity in children [9]. Table 3 lists the targeted constructs and summarizes the curriculum for each intervention component.

#### 2.3.1. Family Psychoeducational Groups

Family psychoeducational groups (FL and FL + FD) were held in the evenings at elementary school buildings or community centers during the spring of children’s 1st grade year. Each family psychoeducational group was facilitated by one graduate and one advanced undergraduate student, one from the field of nutrition and one from either human development and family science or psychology. Concurrent parent and child groups were conducted in separate rooms. Childcare, snacks, and a small participant payment were offered to participating families. The psychoeducational groups included a total of 12 weekly, 90-min sessions (see Table 3).

The Family Lifestyle (FL) component was based on the interventions designed by Epstein and Squires [24] and Satter [25], with a focus on developing healthy food and exercise habits to promote a healthy weight in participating children [26]. Parents and children met in separate groups for the first half of FL sessions and, during the second half, parents and children came together to make and eat a healthy snack. For the Family Lifestyle and Family Dynamics (FL + FD) groups, the first half mirrored the first half of the FL intervention sessions. However, in the FL + FD groups, parents and children remained separate during the second half and participated in the FD content. The FD component focused on general parenting and healthy family relationships (parent) and healthy emotion management and problem solving (child). The parent FD component was developed as an adaptation of the Love, Limits, and Latitude program [27].

Treatment fidelity was maximized by following manualized session scripts, conducting weekly staff meetings, and completing an independent review of a sample of session audio recordings. Fidelity was assessed by independent raters who reviewed randomly assigned audio recordings of group sessions (60% of sessions) and who assessed whether each topic in the intervention manual was covered. Across the 12 sessions for the child and parent FL groups, adherence to the manual was 91% and 90%, respectively. Across the 12 sessions for the child and parent FD groups, adherence to the manual was 88% and 90%, respectively.

#### 2.3.2. Peer Group (PG) Intervention

The PG intervention (received in FL + PG and FL + FD + PG conditions) involved implementation of a curriculum developed and piloted by the last author [28]. The intervention was based on the book, *You Can’t Say*, *‘You Can’t Play!’* (YCSYCP, [29]), which promotes teaching children to accept each other by disallowing rejection at school. YCSYCP facilitators were pairs of graduate and undergraduate students who conducted 12, 30-min weekly sessions across the spring semester in participant children’s 1st grade classrooms (see Table 1). All children in the class received the PG component, regardless of whether they were enrolled in the FiSH Project. To support the intervention in the classroom when the project facilitators were not present, teachers were given Paley’s book to read, were oriented to the YCSYCP curriculum by the last author, and were present when the PG intervention sessions were conducted.

#### 2.3.3. Control Group

The control group consisted of children in 1st grade classrooms in schools randomly assigned to the control condition. As was the case for intervention children, anthropometric data were collected during each wave; however, no classroom or family interventions were offered or conducted with children in control schools.

### 2.4. Analytic Plan

As this was a longitudinal controlled trial with randomization at the school level, we utilized a three-level random intercept model to control for clustering at the school and child levels. We utilized intent-to-treat analyses, which include all cases in the analyses, regardless of level of engagement with the intervention. The intent-to-treat approach maintains the integrity of the randomization and of the estimates of treatment effects [30]. Given that the time between waves was not consistent (Waves 1 and 2 were within the same school year, but Waves 3–5 were annual assessments), we coded the linear effect of time in the model to reflect the time in years from the wave 1 data collection (i.e., Wave 1 = 0, Wave 2 = 0.3, Wave 3 = 1.3, Wave 4 = 2.3, and Wave 5 = 3.3). We also examined both baseline BMI%ile group and intervention condition as moderators of the linear effect of time on the two BMI outcomes. This allowed us to test three-way interactions evaluating the effect of the interventions on BMI over time according to the BMI%ile group the child belonged to at baseline (Wave 1). The effect size at Wave 5 was calculated by multiplying the BMI%ile group effect over time by 3.3 (the final wave) and dividing by the within-person standard deviation at the final wave (obtained from a one-way ANOVA) which results in a measure interpreted the same as Cohen’s *d* [31].

We coded the intervention groups using dummy coding, resulting in four variables coded “0” or “1” to reflect the five mutually exclusive groups. For example, FL is coded one for children at a school that only received FL and zero otherwise; FL + PG is coded as one for children at a school that received both FL and PG and zero for children in schools that did not receive both FL and PG. Children in the control group schools, therefore, have a zero for all four variables (FL, FL + PG, FL + FD, FL + FD + PG) and are the reference group in the analysis.

## 3. Results

The results suggest that clustering was needed for the analysis (Log-Likelihood ratio test against linear regression model: χ2(2) = 627.54, *p* < 0.01). There were no baseline BMI differences between the control group and any of the intervention groups (see Supplementary Materials Table S1 online), indicating that randomization at the school level resulted in similar groups of children across intervention conditions.

### 3.1. Control Group

The control condition showed an increase in raw BMI over time for the at-risk group (B = 0.14, *p* < 0.001; see Table 4). The other BMI%ile groups did not show a significant difference from the at-risk group in the change over time (overweight: B = 0.00, *p* = 0.99; obese: B = −0.01, *p* = 0.82) or from each other (B = −0.01, *p* = 0.82). Thus, in the analyses with raw BMI as outcome, the control group increased in BMI over time regardless of BMI%ile group. The analyses of BMI-M% showed an increase over time for the at-risk group (B = 2.22, *p* = 0.00). The overweight group did not show a significantly higher rate of increase in BMI-M% compared to the at-risk group (B = 1.03, *p* = 0.17). The obese group did show a higher rate of increase over time compared to the at-risk-but-non-overweight group (B = 2.27, *p* = 0.01), but the obese and overweight groups did not differ in increase over time (B = 1.25, *p* = 0.13). Thus, all control children increased in BMI with age, as confirmed by results for raw BMI, but they did not remain at the same distance from median BMI, as confirmed by the disparate results for BMI-M% (see Table 4).

### 3.2. Intervention Effects

The results show the intervention effect over time was moderated by initial BMI%ile group (see Table 5, group difference between treatment group and control group are presented). Children in the at-risk group did not show any significant program effects for any of the treatment combinations. However, the FL and FL + FD treatments showed small effects sizes (−0.20 and −0.28, respectively) for raw BMI, suggesting the potential for favorable effects. The rest of the effect sizes were smaller than 0.20 (a small effect size [31]).

Like the children in the at-risk group, children in the overweight group did not show any significant differences between the treatment combinations and the control group. For the overweight group, no effect sizes were larger than 0.20.

However, children in the obese group who received the FL + FD + PG intervention showed decreased rates of BMI gain compared to controls for both raw BMI (B = −0.05) and BMI-M% (B = −2.36). We also saw a significant effect for FL for raw BMI (B = −0.05), but not mirrored in BMI-M%. All intervention groups except one showed effect sizes of −0.20 or greater for BMI-M% and half the groups for raw BMI, suggesting all interventions reduced weight gain for the obese group. The effect size results for BMI-M% suggest that FL impacts weight gain, but that the effect of all three combined almost doubles the size of that effect.

**Figure 2:**
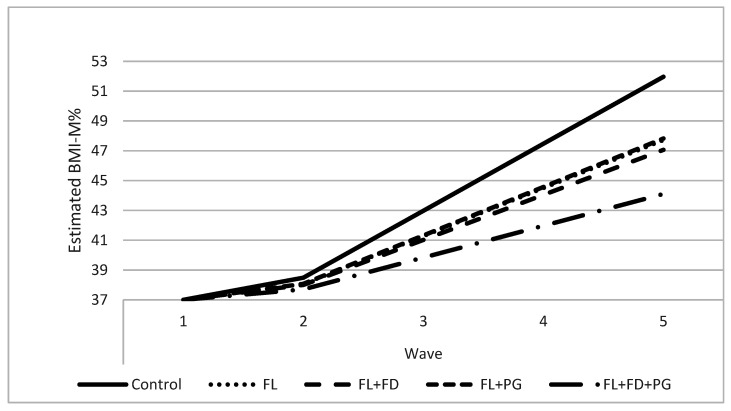
Additive Program Effect on BMI-M% for the Obese Group Over the Five Waves of the Study. Note: Starting point is shown to be the same here to highlight program effects. Estimated BMI-M%.

Figure 2, showing weight trajectories in BMI-M% by treatment for the obese group, further illustrates this combined effect. We see that any form of FL is better than the control (the line for FL alone is almost overlapping with the FL + PG line), but that the combination of all three interventions is a clear improvement over the other treatments.

## 4. Discussion

The present study was a longitudinal cluster, randomized controlled trial of a multi-arm intervention targeting child overweight and obesity. Results showed differential impacts for children as a function of baseline weight status groups. For children who were obese in 1st grade—arguably the group of most health concern—two intervention conditions (FL and FL + FD + PG) significantly lowered weight gain relative to the control group for at least one of the two BMI outcomes. The importance of the combined contribution of all intervention components is evident in Figure 2, suggesting the value of including all three components.

Notable in the current results is the clear difference between the impact of the FL and FL + FD + PG interventions on children who are obese in contrast to the absence of impact on the at-risk (but non-overweight) and children who are overweight. Recent intervention studies [32,33,34], systematic reviews [17,35,36], and umbrella reviews [37] have all stressed the effectiveness of parent-only and parent–child interventions to treat child obesity. The current intervention study shows that effects of traditional family lifestyle interventions that feature nutrition and physical activity are enhanced by adding other family components and, importantly, a school intervention that focuses on increasing the social acceptance of each other by all the children in a classroom. Our family lifestyle intervention had a significant impact on change in raw BMI three years after the intervention. The family lifestyle intervention, when accompanied by our family dynamics intervention that emphasized praise, encouragement, healthy emotion coping and expression, and problem solving, and our in-class intervention that emphasized children’s role playing and discussion of accepting others had a significant impact not only on change in raw BMI, but also change in BMI-M% three years after the intervention. Showing similar impact on both raw BMI and BMI-M% outcome metrics underscores the robustness of the finding. It also emphasizes that significance occurs in a metric (BMI-M%) that is accurate for the children in our study above the 97th percentile in BMI (which BMIz is not) and is the best alternative to BMIz both for children of many ages and for long-term follow-up investigations of intervention studies [23].

Unique contributions of this study include the examination of the relative effectiveness of intervention components targeting proximal (family lifestyle—diet quality and activity) and distal (family dynamics—general parenting and healthy emotion management—and peer group dynamics—social inclusion) influences on weight in children. To the best of our knowledge, no other studies have included all three treatment components and examined the treatments separately by child baseline weight group (at-risk-but-non-overweight, overweight, and obese) longitudinally. Other strengths of the study include three-year post-intervention follow-up data with annual observations, examination of two different BMI outcomes, and use of a non-clinical sample with a relatively high American Indian representation (18%). The focus of this paper was on whether we could find evidence for the intervention effect on weight change. To that purpose, we focused on treatment differences relative to the control group for child BMI. It will be important in future research to examine the relative effectiveness of each individual treatment component and also to examine treatment effects on other health and psychosocial outcomes.

### Limitations

One limitation of the study was the inability to evaluate more definitively the relative effectiveness of each intervention component. In addition, the sample was drawn from relatively small communities in a rural state, limiting the generalizability of findings. However, this is also a strength of the study as few studies have examined the impact of prevention and intervention efforts among rural community samples, where obesity rates are increasing most rapidly [38]. Discrepancies in dosage between conditions is another limitation. Although dosage was the same between the FL and FL + FD components, no comparison intervention was provided for children not participating in the peer group component. As a result, children participating in the PG intervention received 12 more h of intervention than children who did not participate in the PG intervention. Finally, although attrition was not related to child anthropometric measurement, the rate of attrition across the waves of the study is a limitation and may affect the generalizability of study findings.

## 5. Conclusions

In conclusion, a combination of treatment components—psychoeducation about healthy diet and physical activity (FL), psychoeducation about healthy parent–child relationships and healthy emotion management (FD), and a classroom peer acceptance program (PG)—was most effective in reducing child weight gain among children in the obese weight status category. Although the current study did not focus solely on American Indian children and families, its findings add to the literature on interventions for childhood obesity that include this population [39]. The results of this study highlight the importance of a multipronged approach to child obesity treatment that attends to multiple contexts (individual, family, school). In addition to supporting peer group interventions, school systems offer a unique opportunity to engage families in treatment with existing relationships often already in place for recruitment, and convenient and familiar in-community locations for services. Policy makers and treatment providers should continue to identify funding to support school-based intervention programs that engage multiple contexts for children.

## Figures and Tables

**Table 1:** Sample Demographics.

	Total *N*	Intervention		Control		Total	
		*M*	*SD*	*M*	*SD*	Total *M*	Total *SD*
Raw BMI ^a^	538	19.4	3.1	19.1	2.49	19.4	3.0
		N	Percent	N	Percent	Total	Total %
Sex	538	–	–	–	–	–	–
Boys	–	232	50.8%	46	56.8%	278	51.7%
Girls	–	225	49.2%	35	43.2%	260	48.3%
Ethnicity	529	–	–	–	–	–	–
Euro-American	–	322	71.6%	59	74.7%	381	72.0%
American Indian	–	89	19.8%	9	11.4%	98	18.5%
Latino	–	18	4.0%	6	7.6%	24	4.5%
African American	–	10	2.2%	3	3.8%	13	2.5%
Multiethnic	–	9	2.0%	2	2.5%	11	2.1
Other	–	1	0.2%	0	0%	1	0.2%
Maternal Education	187	–	–	–	–	–	–
College Degree	–	55	34.8%	8	27.6%	63	33.7%
Marital Status	212	–	–	–	–	–	–
Married/Remarried	–	141	79.2%	22	64.7%	163	76.9%

Note. ^a^ Raw BMI is true raw BMI adjusted for age and sex. Total analysis sample = 538. No significant differences found between groups on any demographic.

**Table 2:** Percent of Children in each BMI Group for Each Condition at Wave 1.

	FL	FL + FD	FL + PG	FL + FD + PG	Control	Total
At-Risk	28.21%	27.59%	25.00%	27.13%	29.63%	27.32%
Overweight	41.88%	44.83%	29.84%	35.66%	39.51%	37.73%
Obese	29.91%	27.59%	45.16%	37.21%	30.86%	34.94%
Total	100.00%	100.00%	100.00%	100.00%	100.00%	100.00%

Note. At-risk = >75th percentile and <85th percentile, Overweight = >85th percentile and <95th percentile, Obese = >95th percentile. FL = family lifestyle, FD = family dynamics, PG = peer group.

**Table 3:** Summary of the Families and Schools for Health Intervention Components.

Risk Factors Targeted by Intervention Component ^a^	Intervention Curriculum			
**Family Food and Lifestyle (FL) Intervention (12 Weekly Sessions)**				
**Factors Addressed by FL Content**: Interpersonal Context Poor Family Nutritional Intake Low Family Activity Parental Misperception of Child Weight Parent Pressure, Over-Monitoring of Child Eating Intrapersonal Child Mediators Child Emotional and External Eating Dysregulated eating	**First 45 min**		**Second 45 min**	
	Parent Group Traffic light Portion size Red foods Daily activity Introducing foods Healthy snacks Dairy, fruits, veggies Eating out Special occasions Finding balance Healthy child weight Review	Child Group Structure and rules Traffic light Red foods Being active Hungry and full Trying new foods Dairy, fruits, veggies Healthy snacks Dance as activity Review food colors Active games Review	Parent Group Traffic light, continued Portion size, continued	Child Group Structure and rules, continued Traffic light, continued
			Parents and Children Together 3–12: Make and eat a healthy snack	
**Family Food and Lifestyle + Family Dynamics (FL + FD) Intervention (12 Weekly Sessions)**				
**Factors Addressed by FD Content**: Interpersonal Context Poor Parent-Child Communication Discomfort with Negative Emotion Parent Over-Control or Permissiveness Intrapersonal Child Mediators Poor emotional awareness and regulation Poor behavioral self-control Poor Self-Esteem Poor Body-Esteem Loneliness, Depression Anxiety Eating as coping	**Parent Group**		**Child Group**	
	FL Content (45 min) Traffic light Portion size Red foods Daily activity Introducing foods Healthy snacks Dairy, fruits, veggies Eating out Special occasions Finding balance Healthy child weight Review	FD Content (45 min) None None FD introduction Importance of love Child-centered play Praise, Encouragement Emotion coaching Validation Problem-solving Setting limits Effective consequences Wrap up	FL Content (45 min) Structure and rules Traffic light Red foods Being active Hungry and full Trying new foods Dairy, fruits, veggies Healthy snacks Dance as activity Review food colors Active games Review	FD Content (45 min) Structure and rules Valuing uniqueness Things I feel Recognize feeling Showing feelings Expressing feelings Avoiding neg. think Changing neg. think Anger Worry and anxiety Problem solving Review; Wrap up
**Peer Group (PG) Intervention**				
**Factors Addressed by PG Content:** Interpersonal Context Negative Peer Dynamics Peer Exclusion and Dislike Peer Teasing Peer Bullying Intrapersonal Child Mediators Child Negative Affect Poor Self-Esteem Poor Body-Esteem Loneliness, Depression Anxiety Social Avoidance	Weekly Session Topics (Presented to class at school, 12 weeks) A. Introduction to the YCSYCP Principles & Rule ^b^ Meet Magpie Read fairytale, part 1; discussion of YCSYCP Read fairytale, part 2; discussion of YCSYCP Read fairytale, part 3; discussion of YCSYCP Read fairytale, part 5; discussion of YCSYCP Read fairytale, part 6; discussion of YCSYCP YCSYCP rule becomes classroom rule. B. Learning to use YCSYCP 8. Troubleshooting, role playing, discussion 9. Troubleshooting, role playing, discussion 10. Troubleshooting, role playing, discussion 11. Troubleshooting, role playing, discussion C. Don’t Laugh at Me 12. Troubleshooting, book and song, discussion D. Wrap up 13. Troubleshooting, Magpie craft			

Note. Four intervention conditions are comprised of combinations of FL, FD, and PG components: FL, FL + FD, FL + PG, and FL + FD + PG. ^a^ Risk factors identified in the interpersonal and intrapersonal risk model of child obesity [9]. ^b^ You Can’t Say You Can’t Play intervention. Bold and underline are used for headings to improve clarity.

**Table 4:** Control Group Baseline and Change Differences in BMI Outcomes.

	Raw BMI ^a^			BMI-M% ^b^		
	*B*	SE	*p*	*B*	SE	*p*
Baseline for at-risk	1.60	0.05	0.00	9.21	1.90	0.00
Difference in Baseline: At-risk vs. Overweight	0.25	0.06	0.00	7.90	2.48	0.00
Difference in Baseline: At-risk vs. Obese	0.70	0.06	0.00	26.36	2.64	0.00
Difference in Baseline: Overweight vs. Obese	0.46	0.06	0.00	18.46	2.46	0.00
Change for at-risk	0.14	0.02	0.00	2.22	0.56	0.00
Difference in Change: At-risk vs. Overweight	0.00	0.02	0.99	1.03	0.74	0.17
Difference in Change: At-risk vs. Obese	−0.01	0.03	0.82	2.27	0.86	0.01
Difference in Change: Overweight vs. Obese	−0.01	0.02	0.82	1.25	0.82	0.13

Note. ^a^ Raw BMI is log transformed for skew. ^b^ BMI-M% is the log percent distance from median BMI. The test of *p* is a basic z-test for B/SE.

**Table 5:** Comparing Intervention Slope Differences to Controls with Effect Size at the Final Wave.

		Raw BMI ^a^				BMI-M% ^b^			
		Intervention Condition Effects							
Baseline BMI%tile Group		*B*	SE	*p*	ES	*B*	SE	*p*	ES
At-risk	FL vs. Control	−0.03	0.02	0.18	−0.20	−0.75	0.71	0.29	−0.12
	FL + FD vs. Control	−0.04	0.02	0.09	−0.28	−0.87	0.78	0.26	−0.14
	FL + PG vs. Control	0.00	0.02	0.97	−0.01	0.05	0.72	0.95	0.01
	FL + FD + PG vs. Control	0.01	0.02	0.66	0.07	0.66	0.71	0.35	0.10
Overweight	FL vs. Control	0.01	0.02	0.59	0.07	0.18	0.63	0.77	0.03
	FL + FD vs. Control	0.03	0.02	0.15	0.19	1.04	0.62	0.10	0.16
	FL + PG vs. Control	0.02	0.02	0.25	0.16	0.80	0.67	0.23	0.13
	FL + FD + PG vs. Control	−0.01	0.02	0.58	−0.07	−0.55	0.60	0.36	−0.09
Obese	FL vs. Control	−0.05	0.02	0.03	−0.36	−1.27	0.78	0.10	−0.20
	FL + FD vs. Control	−0.02	0.02	0.37	−0.17	−1.47	0.85	0.08	−0.23
	FL + PG vs. Control	−0.02	0.02	0.44	−0.12	−1.24	0.75	0.10	−0.20
	FL + FD + PG vs. Control	−0.05	0.02	0.04	−0.34	−2.36	0.75	0.00	−0.37

Note. ^a^ Raw BMI is log transformed for skew. ^b^ BMI-M% is the log percent distance from median BMI. All estimates from the multilevel models run, not individual estimates. FL = family lifestyle, FD = family dynamics, PG = peer group. The test of *p* is a basic z-test for B/SE.

## Data Availability

The data presented in this study are available on request from the corresponding author. The data are not publicly available due to ongoing data collection.

## Supplementary Materials

The following are available online at https://www.mdpi.com/article/10.3390/ijerph18168744/s1, Table S1: Full Table of Results for Dummy Coded Intervention Conditions.
