# Supplementary material for: The Families and Schools for Health Project: A Longitudinal Cluster Randomized Controlled Trial Targeting Children with Overweight and Obesity
Source: Int J Environ Res Public Health. 2021 Aug 19;18(16):8744. doi: 10.3390/ijerph18168744 (PMC8393339; doi:10.3390/ijerph18168744)
Supplement: Supplementary file 1 [file ijerph-18-08744-s001.zip › ijerph-1283273-supplementary.pdf]

Supplemental Table S1. Full Table of Results for Dummy Coded Intervention Conditions

	[a] Raw BMI			[b] BMI-M%		
	B	SE	*p*	*B*	SE	*p*
Intercept	1.60	0.05	0.00	9.21	1.90	0.00
Wave	0.14	0.02	0.00	2.22	0.56	0.00
Overwt.	0.25	0.06	0.00	7.90	2.48	0.00
Obese	0.70	0.06	0.00	26.36	2.64	0.00
FL	−0.03	0.06	0.57	−0.56	2.50	0.82
FL+FD	−0.01	0.06	0.82	−0.01	2.68	0.99
FL+PG	−0.01	0.06	0.82	−0.11	2.54	0.96
FL+FD+PG	0.01	0.06	0.81	0.54	2.47	0.83
FL X Wave	−0.03	0.02	0.18	−0.75	0.71	0.29
FL+FD X Wave	−0.04	0.02	0.09	−0.87	0.78	0.26
FL+PG X Wave	0.00	0.02	0.97	0.05	0.72	0.95
FL+FD+PG X Wave	0.01	0.02	0.66	0.66	0.71	0.35
Overwt. X Wave	0.00	0.02	0.99	1.03	0.74	0.17
Obese X Wave	−0.01	0.03	0.82	2.27	0.86	0.01
Overwt. X FL	0.00	0.08	0.97	−0.33	3.24	0.92
Obese X FL	0.08	0.08	0.33	2.90	3.46	0.40
Overwt. X FL+FD	0.00	0.08	0.99	−0.21	3.44	0.95
Obese X FL+FD	−0.02	0.09	0.81	−0.98	3.75	0.79
Overwt. X FL+PG	−0.07	0.08	0.38	−2.37	3.34	0.48
Obese X FL+PG	−0.01	0.08	0.90	−0.30	3.34	0.93
Overwt. X FL+FD+PG	−0.03	0.08	0.68	−0.98	3.23	0.76
Obese X FL+FD+PG	0.07	0.08	0.41	4.30	3.34	0.20
Overwt. X FL X Wave	0.04	0.03	0.17	0.93	0.95	0.33
Obese X FL X Wave	−0.02	0.03	0.49	−0.53	1.06	0.62
Overwt. X FL+FD X Wave	0.06	0.03	0.03	1.91	1.00	0.06
Obese X FL+FD X Wave	0.02	0.03	0.63	−0.60	1.15	0.60
Overwt. X FL+PG X Wave	0.02	0.03	0.42	0.75	0.98	0.45
Obese X FL+PG X Wave	−0.02	0.03	0.59	−1.29	1.04	0.21
Overwt. X FL+FD+PG X Wave	−0.02	0.03	0.49	−1.21	0.93	0.19
Obese X FL+FD+PG X Wave	−0.06	0.03	0.07	−3.02	1.04	0.00
Random-effects Parameters	SD	SE	—	SD	SE	—
School Level Intercept	0.03	0.02	—	0.63	1.55	—
Student Level Intercept	0.18	0.01	—	8.22	0.30	—
Residual	0.17	0.00	—	5.61	0.10	—

Note. [a] Raw BMI is log transformed for skew. [b] BMI-% is s the log percent distance from median BMI. FL = family lifestyle, FD = family dynamics, PG = peer group. The test of p is a basic z-test for B/SE.
